# An Innovative Approach to Designing Digital Health Solutions Addressing the Unmet Needs of Obese Patients in Europe

**DOI:** 10.3390/ijerph18020579

**Published:** 2021-01-12

**Authors:** Roberta Patalano, Vincenzo De Luca, Jess Vogt, Strahil Birov, Lucia Giovannelli, Giuseppe Carruba, Claudia Pivonello, Veli Stroetmann, Maria Triassi, Annamaria Colao, Maddalena Illario

**Affiliations:** 1 Dipartimento di Medicina Clinica e Chirurgia, Sezione di Endocrinologia, Università Federico II di Napoli, via S. Pansini n.5, 80131 Naples, Italy; robipatalano@gmail.com (R.P.); cpivonello@gmail.com (C.P.); colao@unina.it (A.C.); 2 Dipartimento di Sanità Pubblica, Università degli studi di Napoli Federico II, via S. Pansini n.5, 80131 Naples, Italy; ricercaesviluppo.diraup@unina.it (V.D.L.); maria.triassi@unina.it (M.T.); 3 UOS Ricerca e Sviluppo, Azienda Ospedaliera Universitaria Federico II, via S. Pansini 5, 80131 Naples, Italy; 4 Empirica Gesellschaft für Kommunikations- und Technologieforschung mbH, Oxfordstr. 2, 53111 Bonn, Germany; jess.vogt@empirica.com (J.V.); strahil.birov@empirica.com (S.B.); veli.stroetmann@empirica.com (V.S.); 5 Servizio di Internalizzazione e Ricerca Sanitaria (SIRS), Unità di Staff Risk Management e Qualità, Direzione Generale, Azienda Ospedaliera Universitaria Policlinico “Paolo Giaccone”, 90127 Palermo, Italy; luciagiov@gmail.com; 6 Servizio di Internalizzazione e Ricerca Sanitaria (SIRS), Azienda di Rilievo Nazionale ad Alta Specializzazione, SIRS ARNAS-Civico, 90127 Palermo, Italy; giuseppe.carruba@arnascivico.it; 7 UNESCO Chair for Health Education and Sustainable Development, Federico II University, 80131 Naples, Italy

**Keywords:** digital health, nutrition, obesity, obese patients, healthy life, healthy food, Europe

## Abstract

According to the World Health Organization (WHO), the worldwide obesity rate has tripled since 1975. In Europe, more than half of the population is overweight and obese. Around 2.8 million people die each year worldwide as a result of conditions linked to being overweight or obese. This study aimed to analyze the policies, approaches, and solutions that address the social and health unmet needs of obese patients, at different levels, in order to simulate the definition of an integrated approach, and to provide and share examples of innovative solutions supporting health promotion, disease prevention, and integration of services to improve the collaboration between the different health and care stakeholders involved across the country and in the lives of obese patients. A collaborative approach involving various levels of government and regional experts from different European countries was applied to identify, explore, and evaluate different aspects of the topic, from the innovation perspective and focusing on a European and a regional vision. Currently, people prefer more foods rich in fats, sugars, and salt/sodium than fruits, vegetables, and fiber. This behavior leads to a significant negative impact on their health-related quality of life. Changes in healthcare systems, healthy policy, and approaches to patient care and better implementation of the different prevention strategies between all the stakeholders are needed, taking advantage of the digital transformation of health and care. Such changes can support obese patients in their fight against an unhealthy lifestyle and at the same time reduce healthcare costs.

## 1. Introduction

Obesity has become a global challenge, and its prevalence has tripled worldwide since 1975 [1]. Overweight and obesity are associated with increased morbidity, mortality, and healthcare expenditures, with a significant negative impact on health-related quality of life [2,3,4]. It has been estimated that, in 2016, 1.9 billion adults 18 years or older were overweight, of whom over 650 million were obese. Additionally, at least 2.8 million people die each year worldwide as a result of overweight or conditions related to obesity [5]. Initially considered only a problem for high-income countries, overweight and obesity are dramatically increasing in low- and middle-income countries too, particularly in urban settings.

In Europe, more than half of the population is overweight and obese [6]. The World Health Organization (WHO) and the World Obesity Federation (WOF) define overweight and obesity as a chronic, relapsing, progressive, and multifactorial disease related to several conditions characterized by an abnormal or excessive fat accumulation with a dramatic impact on economic and social costs [7,8,9,10] in terms of both healthcare expenditures and premature morbidity and mortality [4].

Obesity is caused by an energy imbalance due to a higher intake compared to energy expended [10]. People prefer “energy-dense” foods and drinks, associated with modest or poor physical activity [11]. Paradoxically, an obese patient presents a clinical picture of malnutrition or nutrition deficiencies because of the effect of the so-called “obesogenic diet” rich in refined sugar, saturated fat, and sodium, deficient in folate, copper, iron, calcium, protein, and vitamins [7], and decreased muscle mass, weakness, edema, and/or an impaired immune response [12,13,14,15]. Lower levels of Vitamin A, B, and C are associated, for example, with moderately elevated C-reactive protein (CRP) concentration, which means a higher inflammation status, eventually leading to the development of different chronic diseases (CDs) [16,17]. Moreover, low levels of Vitamin D (Vit. D), very common in obese patients with prevalence rates as high as 90% [7,8], correlate with the incidence of type 2 diabetes mellitus (T2DM). Micronutrient deficit such as lower blood iron, zinc [18,19,20], and magnesium [21,22,23] levels could represent the possible cause for some clinical conditions such as anemia (45% of obese patients) [24,25,26], metabolic syndrome [27], or a reduced magnesium intestinal absorption [17] as demonstrated in some studies in obese patients before bariatric surgery [27,28,29].

Overweight and obesity have a high impact on multiple physical and psychological parameters. Chronic stress, as well as discrimination, play a role not only in the development but also in the persistence of obesity [30], and it has a deep psychological and physical impact resulting in eating behavior changes, psychosocial stress, and indirect effects on social relationships [31,32]. Some researchers have defined obesity as a “neuropsychological disease” [33] because, apart from the impact on the impairment of physical functioning, health perceptions, and vitality, the scientific evidence suggests a link with mental disorders such as depression, anxiety, panic disorders, or mood disturbances. In turn, psychiatric disorders can also cause obesity as a result of drug effects or hormone imbalances, leading to mental illness as a result of poor self-image and physical illnesses [34,35,36,37]. Indeed, a history of mental illness may increase the risk of developing obesity, while obesity may increase an individual’s chances of developing a psychiatric disorder [38]. Men and women present a different link between mental health and obesity [39,40]. Obese adolescents and adult females have a higher probability of developing depression than obese male counterparts [41,42,43,44]. Surprisingly, some interesting data have demonstrated that symptoms of depression, anxiety, and stress started to drop significantly after adopting a Mediterranean lifestyle for three weeks [45], or participants stated they felt better emotionally and physically after participating in dietary counseling sessions that made them feel they had better control over their disease [46].

Several studies provide clear indications that all Western countries, including Italy, are witnessing real epidemics of adult and childhood obesity and non-communicable diseases (NCDs), mostly because of the dramatic changes that have occurred in both food systems and consumer eating habits and behaviors globally [47,48]. A balanced healthy diet is not the same for every individual—while the principles may be the same, several factors influence the results, such as age, gender, lifestyle and degree of physical activity, cultural context, locally available foods, and dietary habits [1,49,50,51]. In this framework, the Mediterranean lifestyle could represent an example of an effective approach to counteract obesity and might be effective in the treatment of some chronic diseases such as diabetes, metabolic syndrome, and cardiovascular diseases [52,53,54,55,56,57,58,59,60,61].

Lifestyle modification programs based on weight loss interventions, self-monitoring, and coaching represent the first-line approach in the treatment of obesity [62,63]. These programs however are not always applicable and are difficult to use because of the time required to run the programs, lack of expertise from healthcare providers, the financial burden to patients, or issues with the insurance companies [64].

Digital health solutions have the potential to represent a new tool to manage patients on health interventions through mobile apps, wearable devices, telemedicine, or video games [65,66,67]. Mobile apps can promote a healthy lifestyle, encourage individuals to be healthier and more active, and offer smartphone-based physical activity coaching interventions. Indeed, users started to feel better and healthier by using them [66], suggesting that digital health solutions are capable of improving physical activity levels [67]. In this highly fragmented and complicated context, there is an urgent need to develop appropriate policies, programs, and campaigns for nutritional counseling and education of citizens based on scientific and solid evidence and shared verifiable information. The Mediterranean diet (MD) could thus represent a primary food approach that is tailored to the local sociocultural context and may represent a sustainable intervention to integrate with service providers for specific persona types.

The objective of this study was to analyze the policies, approaches, and solutions that address the social and health unmet needs of obese patients, at different levels, in order to simulate the definition of an integrated approach, taking into consideration the experiences already adopted, and to provide and share examples of innovative solutions supporting health promotion, disease prevention, and integration of services to improve the collaboration between the different health and care stakeholders involved across the country and in the lives of obese patients.

## 2. Methodology

A qualitative approach was adopted in the current study to identify and specify key digital solutions and high-impact user scenarios in Active and Healthy Ageing (AHA). In order to achieve a successful outcome, an iterative approach was adopted to develop a user-centered perspective for the identification of solutions addressing health needs with different complexity, expressed along the entire life course. A collaborative effort was undertaken, to ensure that experts with multidisciplinary expertise and representatives of all stakeholders in the field of the digital transformation of health and care for active and healthy aging were involved. This ensured that their perspectives were represented in the development of the persona matrix and profiles, thus facilitating the identification of suitable solutions, more likely to be accepted and successful. The stakeholders involved included various levels of government and regional experts from different European countries, including European policymakers, regional authorities, industrial players, health professionals and civil society organizations, and multistakeholder platforms such as the European Innovation Partnership on Active and Healthy Ageing (EIP on AHA), belonging to the European project WE4AHA of which the Blueprint on Digital Transformation of Health and Care for the Ageing Society is a key output [68].

Four initial key topic areas were chosen, which were identified jointly by European Partners on both the demand and supply sides of the market:

1. Data analytics for predictive risk stratification and prevention;
2. Proactive prevention through empowerment, self-management, monitoring, and coaching;
3. Digital solutions for connected health;
4. Digital support for integrated care.

These four categories of digital tools were used to compile a compendium of the different digital solutions available that may meet the needs of varied population segments defined by both age and the complexity of their health status. This compendium was assembled through a general consultation, several informal multidisciplinary and multistakeholder focus groups, and two workshops with groups of representatives from the variety of fields described above, taking place in the period November 2017—November 2020 as part of events organized within the framework of the EIP on AHA (“the Partnership”). With the Partnership’s reach to over 1000 stakeholders across Europe, this approach ensured inclusiveness when collecting inputs from a diversity of European geographical and cultural backgrounds.

To strengthen the widest involvement possible (beyond inputs of event attendees), in a second step data were collected via a survey, mainly targeting the industry representatives in the Partnership as to how well this compendium of digital solutions best met the needs of the various population segments represented by personas. In all, 70 survey responses were submitted by health tech industry representatives of both large and small and medium enterprises (SMEs), providing information about their existing good practices and how they match the needs of the population segments.

Data were compiled by the Blueprint Partners into a detailed picture of the different needs in Europe that were addressed by the identified innovative good practices. The overview was shared with the EIP on AHA partners and welcomed by demand-side stakeholder groups (e.g., healthcare providers), to better understand the needs of their populations, and by supply-side stakeholder groups (e.g., industry), who can inform the development of their innovative practices and tailor them better to the expectations of the demand side. An overview of key information and telecommunication technology (ICT)-enabling technologies available and barriers to scaling up is also provided, in order to promote innovation, make health and care systems sustainable, and achieve economic growth in Europe.

The Blueprint approach to identify high-impact user scenarios in AHA is adopted in different settings, such as in the software industry and the health and care sectors. With this approach, innovative solutions are designed based on how a user from a target group will interact with technologies [69] by starting to develop “personas”. A persona is defined as a single, specific hypothetical/fictitious person who represents a segment of the population [70] with a realistic name, a face, and a description of their character (needs, goals, hopes, dreams, and attitudes). The Blueprint persona descriptions, developed by the Blueprint Partners, include also behavioral characteristics, which could affect both short-term and long-term success with interventions directed toward managing a disease or adopting wellness [71,72], for example, a persona’s trust or lack of trust in care professionals, their self-management capabilities, and specific details about their character (e.g., being prone to aggressive behavior or having the tendency to reject outside support). Twelve different personas have been developed by the Blueprint Partners, which represent different “population segments”. They have been classified according to age or life-course groups (children/young adults, working age adults, retired persons below 80, persons aged 80+) and their corresponding well-being as well as health and care needs (good well-being, chronic conditions and/or social needs, complex needs).

**Figure 1:**
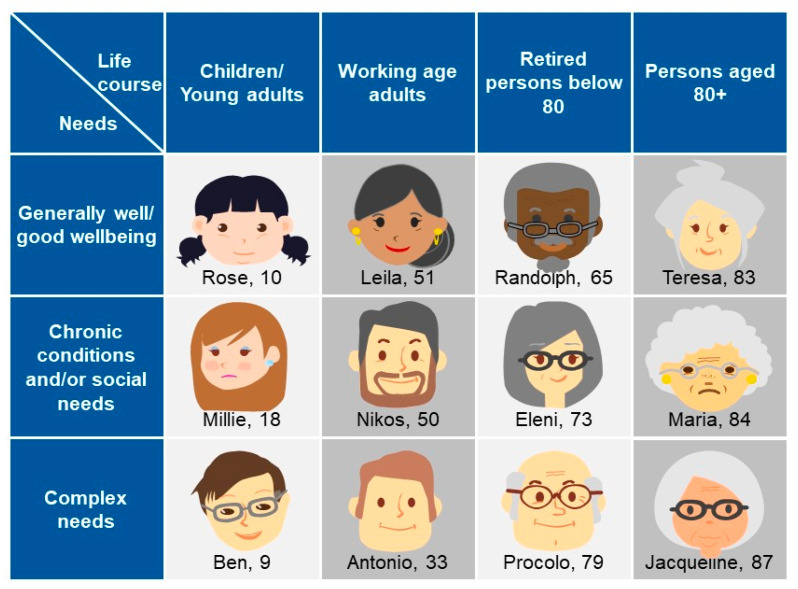
The Blueprint personas developed by Blueprint Partners, representing different needs, aspirations, attitudes, dreams, disease-related characteristics, care needs of certain groups in the society, and identification of what is important to them [68].

**Figure 2:**
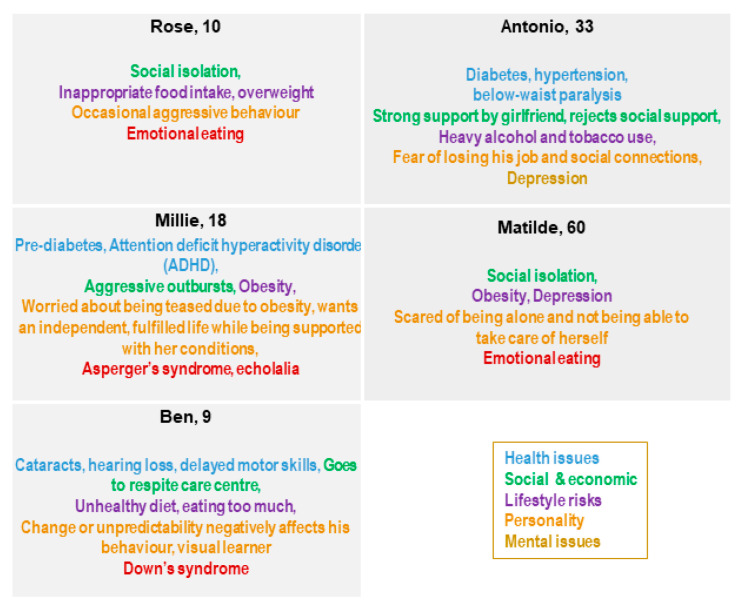
Rose, Millie, Ben, Antonio, and Matilde and their key points [68].

The Blueprint personas aim at facilitating the identification of health needs that may be addressed by integrating innovative solutions in their care and cure. This approach is person-centered, focused on specific target groups to identify what is important to them, taking into account not only health needs but also IT skills, socioeconomic context, interoperability, and integration gaps that may influence the adoption of innovative solutions tailored to improve health outcomes. Of note, overweight or obese patients tend to be marginalized and subjected to a real social stigma [31,73], and digital solutions may be useful to overcome psychological factors that prevent obese patients from starting their journey in a lifestyle change [74]. Indeed, among the twelve personas developed (Figure 1) by Blueprint Partners, five—Rose, Millie, Ben, Antonio, and Matilde developed later (Figure 2)—are overweight or obese due to low physical activity, making them at risk for developing diabetes and becoming socially isolated [68]. They represent different “population segments”, age and life-course groups with different needs and diseases.

Rose is an overweight 10-year-old child who lives in a suburb of a large city where there are not many programs for children to go outside and play, with her classmates making fun of her because of her weight.

Millie, 18 years old, and Ben, 9 years old, are affected by autism spectrum disorder (ASD, specifically Asperger’s Syndrome) and Down’s Syndrome, respectively. Both Millie and Ben live with their family and need to follow a healthy diet to control body weight because of their overweight condition.

Antonio, a 33year-old electrician, is affected by diabetes and lives with his family. He needs to change his lifestyle to be more active and follow a healthy diet to manage diabetes and avoid progressing from a mild overweight to an obese condition. He spends a lot of time at home with his family and girlfriend since he has had a car crash and needs a wheelchair to move around.

Matilde is a 60-year-old woman affected by a severe state of depression that does not allow her to deeply understand her overweight condition.

No need for Medical Ethical Committee approval was identified, as no data were used, neither were references made to a specific single patient. “Persona” is a theoretical elaboration of prototypes representative of a certain typology of a patient.

## 3. Results

### 3.1. Health Policy and Obesity (World Health Organization (WHO), European Commission (EC), National, and Local)

The World Health Organization (WHO) is carrying on new strategies to promote digital health solutions to decrease the obesity rate in all countries by 2025 [75]. Despite progress, many countries still require support to develop national digital health strategies. The Global Strategy on Digital Health 2020–2025 aims to enhance the digital health networks, to promote higher standards of health and access services, and to integrate financial, organizational, human, and technology resources for the best use of digital services [76,77]. In this framework, the health-related sustainable development goals (SDGs) and the Thirteenth General Programme of Work (GPW13) play an important role in supporting the development of digital solutions ensuring healthy lives and promoting well-being at all ages [78,79]. Digital health provides concrete and valuable options to enhance health and well-being, supporting the development of national programs to improve healthcare delivery and strategies, and to ensure universal health coverage and availability of good health data and reporting related to SDGs [76,80], and identifies digital services to promote equitable and universal access to health for all related to GPW13 [80].

Furthermore, Europe promotes a smart and inclusive health approach with the European Union (EU)’s 10-year economic growth strategy in order to keep people healthy and active for longer and make the healthcare sector more sustainable [81]. Among the different actions promoted by that strategy, the Innovation Union and Digital Agenda for Europe are the most relevant. The Innovation Union’s objectives are to push for new innovative approaches achieving an Active and Healthy Ageing (AHA) across the European countries [82,83]. Therefore, a pilot European Innovation Partnership on Active and Healthy Ageing was launched in 2011, of which Blueprint on Digital Transformation of Health and Care for the Ageing Society represents a model of application [68,84], aiming to improve the average healthy lifespan in the EU. To achieve this set goal, this pilot is pushing to improve the efficacy and sustainability of the healthcare system and innovative products and services that respond to the aging challenge, and EU citizens’ health literacy to ensure them an AHA [84].

The Digital Agenda for Europe aims to enable the use of digital applications or services across the European countries in order to achieve a better quality of life and care by promoting independent living among sick citizens or those with disabilities and, not less important, to reduce medical costs [81,85]. For this purpose, ensuring online access to their medical health data or attempting to develop telemedicine to foster interoperability and eHealth certification in all the EU’s Member States is a real challenge to achieve [81,85].

In particular, the European Commission [86,87] is trying to respond to the global rise of overweight and obesity by adopting the White Paper on a Strategy for Europe on Nutrition, Overweight and Obesity-related health issues [88], Indeed, the EIP on AHA’s Action Group A3 on Frailty has developed a gradual approach to malnutrition in the elderly population, which links assessment to adequate interventions (primary, secondary, tertiary) and aims to implement innovative tools for effective measures of prevention, detection, and treatment of obesity and malnutrition [89]. Meanwhile, the “Blueprint on Digital Transformation of Health and Care for the Ageing Society” deals with the challenging aspects on how innovation can transform health and care provision in our aging society in key health and care topic areas that benefit from digital transformation using a life-course approach [68].

In Italy, the National Prevention Plan [90] responds to the increased overweight and obesity rate with preventive programs focused on increasing skills in the sector and exchanging experiences through promoting knowledge. The Plan focuses on training activities on the best obesity prevention practices; identifying and disseminating effective methods related to obesity prevention, through a repository based on Evidence-Based Prevention (EBP); and sharing good clinical practices, through a webspace dedicated to the dissemination of the most significant documents in the literature, sharing intervention protocols as well as strategies for evaluating interventions and results, and supporting local prevention interventions.

The National Plan for Chronic Diseases [91], instead, aims to improve the protection of patients with chronic diseases; reduce the burden on the individual, their family, and the social context; improve the quality of life; and make health services more effective and efficient in terms of prevention and assistance and ensure fair access for citizens. This Plan identifies a list of chronic diseases for which there is no specific plan yet available at the national level by using epidemiological studies on their severity, invalidity, welfare and economic weight, the difficulty of diagnosis, and access to treatment. One of the main objectives is to promote a healthy lifestyle in people at risk, to prevent chronic diseases. The Plan is implemented by the different Italian Regions and they are implementing it based on their economic resources.

Among Italian Regions, the Campania Region joined the OKkio health surveillance system [92] in 2008, which aims at assessing and monitoring the evolution of the nutritional situation of primary school children (8 years of age) and their school environment, focusing on correct nutrition and physical activity. The protocol is based on a population surveillance approach carried out in a primary school population. In 2010, the Campania Region joined the PASSI d’Argento (Silver steps) surveillance system [93] focused on a healthy lifestyle in over 64-year-olds with disabilities or at risk of disability. In addition, for all those people at risk of chronic diseases, the Campania Region has developed a preventive plan promoting physical activity [94,95].

In the Sicily Region, the Department of Health (DASOE) has initiated a program called “Formazione Educazione Dieta” (FED), aimed at the promotion and diffusion of healthy lifestyles, according to the MD [96,97]. The major methodological strength of the FED program consists of an intervention of primary disease prevention and health promotion based upon the following two strategic elements: a centralized planning, adjustable according to outcome indicators, and a multi-year action plan, with cyclic activities throughout three regional networks (health, education, and agribusiness). The program is synergistically focused on (a) a cascade training model, aimed at qualifying people to become expert figures of trainers or educators able to impact on the cultural changes, behaviors, and lifestyles of the population, by acting specifically on the various recipients, in accordance with appropriate methodologies and evidence-based contents and (b) the activation of local networks built to promote capillary activities in the regional territory. Using an integrated multi-professional approach, the experts develop an operational and training program with centrality and uniqueness of address, making it uniformly applicable to the distinct organizational realities of the regional territories (Table 1).

### 3.2. Innovative Approaches to Address Obesity: A European Vision

The European Commission is working to provide its citizens access to safe and top-quality digital services in health and care in different ways. The digital transformation of health care will benefit people, healthcare systems, and the economy. Indeed, digital technologies such as 4G/5G mobile communication, artificial intelligence, or supercomputing offer new opportunities to transform health and care service by enabling innovative approaches to independent living or integrated health and social care. Health data and advanced data analytics can help accelerate scientific research, personalized medicine, early diagnosis of diseases, and more effective treatments [83].

The Digital Single Market (DSM) designates the strategy of the European Commission for the best access to the online world for individuals and businesses. A DSM is one in which persons, services, capital, individuals, and businesses can access and engage in online activities under conditions of fair competition, and a high level of consumer and personal data protection, irrespective of their nationality or place of residence [85].

PUBLIC HEALTH is a Best Practice Portal, a “one-stop shop” for consulting good and best practices collected in actions co-funded under the Health Programmes and for submitting practices for assessment [98]. The Directorate-General for Health and Food Safety (DG SANTE) aims to identify, disseminate, and transfer best practices to progress in health promotion and NCD prevention and management in Europe, and to reduce premature mortality from NCDs by one-third by 2030, through prevention and treatment [99]. An example of a good approach to this topic comes from Ireland. Little Bites, a one-stop shop for food safety, food allergen, and healthy eating advice for all early childcare providers, developed in collaboration with Early Childhood Ireland [100], and Safefood [101] represent a hub that provides excellent downloadable resources, recipes, and some lovely videos and photos of children enjoying their dining experiences in Irish early childhood settings (Table 2).

### 3.3. Innovative Approaches to Address Obesity: The Italian Vision

In Italy, the Agency for Digital Italy (AgID) is intended to ensure the achievement of the objectives of the Italian digital agenda, to contribute to the dissemination of information and communication technologies, and to help the uptake of innovation and economic growth [102].

The Digital Healthcare System refers to all the interventions undertaken by central, regional, and local Administrations to promote digital innovation of healthcare processes [103]. The interventions targeted at the digital healthcare system aim to improve healthcare services promoting healthy behavior among patients, ensure health monitoring and disease prevention and management, limit waste and inefficiencies, improve the quality–price ratio of healthcare services, and reduce the differences among regions.

Smart Health, for example, represents an innovative service for home telemonitoring and remote diagnosis support. Using a medical kit available to patients at home, consisting of a device for sending data in real time to the doctor and a set of medical devices (to control blood pressure, blood glucose, or heart rate or a digital pill dispenser capable of issuing alarms and signals, etc.) and a digital platform, it is possible to send their clinical data to medical staff in real time and to be monitored remotely, thereby reducing the inconvenience and costs of travel for visits and controls at health facilities. In this way, it is possible to monitor patient parameters and the effectiveness of treatments [104]. IPPOCRATE AS [105] is a software house specialized in the development of eHealth and smart healthcare solutions. It consists of a set of services and digital tools for health and medical care. The software house uses information and telecommunication technologies (ICTs) to improve activities such as prevention, diagnosis, and treatment, as well as to improve the monitoring and management of health and lifestyles. IPPOCRATE AS is involved in both Italian and European research and development consortia with a long experience in obtaining research funds by drawing on the agencies of the European Commission, Ministries, and Regions.

Move your health provides an integrated path toward the prevention and management of overweight and obesity, promoting healthy lifestyles in the Autonomous Province of Trento. It proposes tools and initiatives aimed at helping families to choose healthier lifestyles and reducing health inequalities associated with outreach activities, involving migrant or socioeconomically disadvantaged families [106] (Table 2).

#### Experiences of Italian Regions: Campania and Sicilia

The Campania Region is committed to supporting the digitization of processes and services in the healthcare sector. Technological innovation represents one of the most important topics in the health policy strategies of the Region to improve the quality level of health services for the citizens in Campania.

The Region’s partner in innovation policies is the “Società Regionale per la Sanità Spa (Soresa)”, an instrumental company set up by the Campania Region to carry out strategic actions in regional health expenditure. “Soresa” is involved in study and research activities on the use of artificial intelligence in health care and is oriented toward the use of Internet of Things (IoT) platforms based on microservices that allow the efficient and fast implementation of data collection and systematization applications in the health sector [107].

In the Sicily Region, the main objective of the Regional Health Department is to provide the entire Regional Healthcare System (RHS) with a strategic plan for the realization of various investments to support the government of the RHS in which, through a systemic approach defined by the “central” level, each project initiative fits coherently and without any overlap in a wider digital innovation framework. In this direction, the basic strategy of the regional plan comprises the full involvement of the different organizational elements that compose the RHS, providing a comprehensive program that defines the reference perimeter, i.e., the documents to address and coordinate operational interventions according to a shared vision of the development of digital healthcare in the Sicily Region to be implemented immediately and pursued in the years to come. A major goal is to procure maximum effectiveness, transparency, and simplicity, both at the level of health professionals and operators and, especially, of the general public and/or other stakeholders. The strategic approach is permeated by the need to establish a single dedicated access point to guarantee the activation of a “dialogue system” with the various categories of users, while allowing the communication to be managed in an effective, streamlined, and timely manner. In particular, the Regional Department of Health aims at implementing a Regional Health Information System (RHIS) addressed to health professionals as an instrument of governance, also allowing, in an evolutionary perspective, the potential integration with other sectors or areas not strictly related to health. This approach achieves multiple objectives, namely the governance of internal processes, the availability of a cognitive framework always updated on the use of resources for planning purposes, and a Citizen Portal, a single point of reference for citizens through which they can have online access to health services, information on organizational aspects, as well as the management of a series of accessory aspects (e.g., ticket payments, choice and revocation, expression of consent [108].

Opera [109], included in the projects carried on by the UNESCO Chair on “Health Education and Sustainable Development”, is part of a great event, Campus 3S [110] (Salute, Sport e Solidarietà), which since 2010 has been moving from Naples to the biggest Italian squares. This project offers an innovative free path through which men and women will be visited and have the possibility to start a program to lose weight. The project is based on the organization of medical, sports, and tasting events and a psychological path to provide a completely free medical examination to overweight or obese patients, including measurement of BMI calculation and waist circumference, assessment of body composition, electrocardiogram (ECG), and blood pressure measurement, spirometry, measurement of the glycemia and eating habit; to evaluate muscle mass through some exercise; and to perform a taste-and-smell sensitivity test to verify their ability to recognize food. They are also invited to taste some ad hoc foods prepared by prestigious chefs and to undergo a psychological examination using questionnaires to evaluate possible areas presenting an obstacle to starting the path.

Cambiovita is a built-in pathway to curing obesity. The program was developed by Cardarelli Hospital, in Naples, in collaboration with the patients’ association “Insieme amici obesi” (Obese friends together). The main role is played by the patient who tells his/her story, exposes his/her doubts, and presents his/her difficulties [111] (Table 2).

## 4. Conclusions

Currently, people consume more foods rich in fats, sugars, and salt/sodium but not enough fruits, vegetables, and fiber [48,49,50]. A healthy and balanced diet is very important in our life, and when combined with physical activity, it is pivotal to achieving and maintaining an adequate weight and a good psychological state, while reducing the risk of developing chronic diseases. Several factors increase the risk of obesity in different settings, such as family, school, community, and workplaces. For these reasons, the WHO’s Member States are carrying out programs to promote healthy diets, trying to act on both social and economic factors such as income, food prices, individual preferences, cultural traditions, and geographical and environmental aspects (including climate change) involving multiple sectors and stakeholders such as government and the public and private sectors.

A central role in creating a healthy food environment is played by governments who enable people to adopt and maintain a healthy lifestyle. Governments achieve this by ensuring coherence in national policies and investment plans, including trade, food, and agricultural policies, by promoting a healthy diet and protecting public health, and by promoting appropriate infant and young child feeding practices.

In all the Blueprint persona cited as examples, social support, development of a health-friendly environment, and educational programs on healthy nutrition and physical activity should be of great help in managing their relationships with food and psychological well-being as well as maintaining positive mental health through social and/or outdoor activities such as developing skills or meditation (Table 2). Public health approaches to reducing the burden of obesity or depression must consider the strong association between these two common conditions. Such an approach facilitates an interdisciplinary and “user-centered” design of digital solutions, allowing for their adaptation. Evidence has emerged that the addition of digital tools may indeed facilitate patient behavior change of an unhealthy lifestyle, integrating positive psychology practices and possibly leading to a larger proportion of patients accessing structured interventions for healthy lifestyle promotion [112,113,114]. The implementation of a persona approach may also be useful for the early involvement of end-users in solutions’ design and adaptation, increasing adherence, and hence the effectiveness of digital solutions [115]. Persona profiles also consider the potential benefits that can be derived from digital resources for the patient and associated stakeholders such as careers (informal or formal), healthcare professionals, and healthcare providers.

Intersectoral and cross-cutting approaches between policies have proven their effectiveness in preventing obesity and reducing related risks for health, while ensuring monitoring and surveillance. Nonetheless, sustainability issues hinder the scale-up of such approaches to impact obesity at the population level [116]. The digital transformation of health and care that obtained a boost due to the Covid-19 pandemic provides several validated and innovative solutions supporting health promotion, disease prevention, and integration of health services that facilitate the collaboration between all involved stakeholders. Nonetheless, there is a need to plan implementation in the framework of a shared vision and toward specific objectives. Artificial intelligence could provide personalized information or recommendations to the patient or citizen, health professionals involved in prevention and care paths, and health authorities to develop targeted intervention strategies. IT integration and interoperability among data systems would allow designing an innovative model for decision support, based on clinical and laboratory results of patient groups, including data derived from self-monitoring (wearable), lifestyle monitoring (nutrition, physical exercise), socialization, drug interactions, and omics data.

The collaborative ecosystem of the EIP on AHA has been supporting the stakeholders’ engagement along a quadruple helix of innovation, which is pivotal to aligning investments between international, national, and international levels. In Italy, the ProMIS network for the internationalization of the regional health system has been a key enabler to speed up large-scale adoption of innovative good practices [117]. At the regional level, the commitment to support the digitization of processes and services in the healthcare sector has been translated into the establishment of dedicated agencies for technological innovation. Still, there is an inadequate structured engagement of the non-profit sector, which is pivotal to ensuring citizen involvement and needs assessment at scale and strengthening the link between public and private sectors. The participatory design of digital solutions in which the end-user is no longer the passive recipient of a new tool but is an integral part of the design and the innovation process would allow to evaluate the care experience that is held only by the patient. Furthermore, a joint effort should be undertaken, between key stakeholders involved in training and education, to ensure the adoption of multidisciplinary approaches that extend their reach toward citizens’ literacy and empowerment [117,118].

## Figures and Tables

**Table 1:** Overview of health policies.

**Health Policy**
**World Health Organization**
**Global Strategy on Digital Health 2020–2025:** ▪ Sustainable development goals (SDGs) ▪ Thirteenth General Programme of Work (GPW13)
**Europe**
**EU’s 10-year economic growth strategy:** ▪ **Innovation Union** o European Innovation Partnership on Active and Healthy Ageing o Blueprint on Digital Transformation of Health and Care for the Ageing Society ▪ **Digital Agenda for Europe**
**White Paper on a Strategy for Europe on Nutrition, Overweight and Obesity-related health issues**
**Italy**
▪ **National Prevention Plan** ▪ **National Plan for Chronic Diseases**
**Campania and Sicily Regions**
▪ **OKkio health surveillance system** ▪ **PASSI d’Argento surveillance system** ▪ **Formazione Educazione Dieta program**

**Table 2:** Digital tools and Blueprint persona unmet needs

Digital Tool	Blueprint Persona
**Europe**	
▪ **Little Bites** ▪ **Safefood**	**Rose, Millie, and Ben**: healthy diet and eating advice to control body weight
**Italy**	
▪ **Smart Health**	**Antonio and Matilde**: home telemonitoring, remote diagnosis support, update data in real-time to the doctor, medical devices to be monitored remotely, reducing the inconvenience and costs of travel for visits and controls at health facilities
▪ **IPPOCRATE AS**	**Rose, Milli, Ben Antonio and Matilde**: improve activities such as prevention, diagnosis, and treatment, as well as improve the monitoring and management of health and lifestyles
▪ **Move your health**	**Rose, Milli, Ben Antonio and Matilde**: choosing healthier lifestyles and reducing health inequalities associated with outreach activities, involving migrant or socioeconomically disadvantaged families
**Campania and Sicily Regions**	
▪ **Opera**	**Rose, Milli, Ben Antonio and Matilde**: innovative free path to men and women who want to lose weight, healthy diet and eating advice to control body weight, psychological support
▪ **Cambiovita**	**Rose, Milli, Ben Antonio and Matilde**: psychological support, the patient himself/herself who tells his/her story, exposes his/her doubts, and presents his/her difficulties

## Data Availability

The data presented in this study are openly available at https://ec.europa.eu/eip/ageing/blueprint_en.
